# Does the Anatomical Type of the Plantaris Tendon Influence the Management of Midportion Achilles Tendinopathy?

**DOI:** 10.3390/jcm14155478

**Published:** 2025-08-04

**Authors:** Łukasz Olewnik, Ingrid C. Landfald, Bartosz Gonera, Łukasz Gołek, Aleksandra Szabert-Kajkowska, Andrzej Borowski, Marek Drobniewski, Teresa Vázquez, Kacper Ruzik

**Affiliations:** 1 Department of Clinical Anatomy, Mazovian Academy in Płock, 09-402 Płock, Poland; ingridceciliee@gmail.com (I.C.L.); b.gonera@mazowiecka.edu.pl (B.G.); l.golek@mazowiecka.edu.pl (Ł.G.); a.szabert-kajkowska@mazowiecka.edu.pl (A.S.-K.); k.ruzik@mazowiecka.edu.pl (K.R.); 2 VARIANTIS Research Laboratory, Department of Clinical Anatomy, Mazovian Academy in Płock, 09-402 Płock, Poland; 3 Department of Orthopaedics and Traumatology, Medical University of Łódź, 90-419 Łódź, Poland; andrzej.borowski@umed.lodz.pl (A.B.); marek.drobniewski@umed.lodz.pl (M.D.); 4 Bodies Donation and Dissection Room Center, Department of Anatomy and Embryology, Faculty of Medicine, Complutense University of Madrid, 28040 Madrid, Spain; tvazquez@ucm.es

**Keywords:** plantaris tendon, plantaris muscle, mid-portion Achilles tendinopathy, Achilles tendinopathy

## Abstract

**Background:** Midportion Achilles tendinopathy (Mid-AT) is a complex condition that may be exacerbated by anatomical variations of the plantaris tendon. Recent anatomical studies, particularly the classification proposed by Olewnik et al., have enhanced the understanding of plantaris–Achilles interactions and their clinical implications. **Objective:** This review aims to assess the anatomical types of the plantaris tendon, their imaging correlates, and the impact of the Olewnik classification on diagnosis, treatment planning, and surgical outcomes in patients with Mid-AT. **Methods:** We present an evidence-based analysis of the six anatomical types of the plantaris tendon and their relevance to Achilles tendinopathy, with emphasis on MRI and ultrasound (USG) evaluation. A diagnostic and therapeutic algorithm is proposed, and clinical outcomes of both conservative and operative management are compared across tendon types. **Results:** Types I and V were most strongly associated with symptomatic conflict and showed the highest benefit from surgical resection. Endoscopic approaches were effective in Types II and III, while Type IV typically responded to conservative treatment. Type VI, often misdiagnosed as tarsal tunnel syndrome, required combined neurolysis. The classification significantly improves surgical decision-making, reduces overtreatment, and enhances diagnostic precision. **Conclusions:** The Olewnik classification provides a reproducible, clinically relevant framework for individualized management of Mid-AT. Its integration into imaging protocols and treatment algorithms may improve therapeutic outcomes and guide future research in orthopaedic tendon pathology.

## 1. Introduction

### 1.1. Anatomy and Function of the Achilles Tendon and the Plantaris Muscle

The Achilles tendon (AT) is the strongest and largest tendon in the human body, formed by the convergence of the gastrocnemius and soleus muscles, inserting into the posterior aspect of the calcaneus. It plays a crucial role in plantarflexion of the foot, enabling efficient gait, running, and jumping activities [1]. Adjacent to the AT lies the plantaris muscle, a slender and variable structure traditionally considered vestigial. However, more recent anatomical investigations have challenged this view, highlighting its potential clinical relevance [2]. The plantaris originates most commonly from the lateral supracondylar line of the femur and courses distally as a long, thin tendon that typically inserts medial to the AT on the calcaneus [3,4,5,6,7]. Notably, the plantaris tendon (PT) may exhibit a wide range of anatomical variations, including multiple origins or unusual insertions, which have been increasingly linked to pathological conditions [3,7].

### 1.2. Definition and Significance of Midportion Achilles Tendinopathy (Mid-AT)

Mid-AT is a common overuse injury characterized by pain, swelling, and impaired function in the tendon region approximately 2 to 7 cm proximal to its insertion on the calcaneus [8,9]. It affects both athletes and the general population and is associated with tendon degeneration rather than acute inflammation [10,11]. The condition is often resistant to conservative treatment, which includes eccentric loading, extracorporeal shockwave therapy, and injections [1]. As such, understanding the underlying anatomical factors that may contribute to persistent or refractory cases is of increasing importance.

### 1.3. The Hypothesis of Plantaris Tendon Involvement as a Biomechanical Factor

In recent years, growing attention has been directed toward the potential role of the PT in the pathogenesis of Mid-AT. It has been hypothesized that its close proximity or even interposition with the medial aspect of the AT may result in a compressive or shearing effect, contributing to localized pain and degenerative changes [12,13]. Histological and imaging studies have provided evidence of pathological changes in the PT in patients presenting with chronic Achilles symptoms, often in the absence of significant Achilles pathology [14,15]. These findings have led some surgeons to incorporate plantaris tendon excision into surgical protocols for recalcitrant Mid-AT, often with favorable outcomes [16,17].

### 1.4. Aim of the Study: Evaluating the Olewnik Classification and Its Clinical Relevance

Given the substantial anatomical variability of the PT, a standardized classification may aid in both diagnosis and surgical planning. Olewnik et al. [3,4] proposed a detailed anatomical classification of plantaris tendon morphology based on cadaveric and imaging studies, identifying multiple types with distinct courses and insertion patterns [3,4]. This review aims to critically evaluate whether such anatomical typing, particularly the Olewnik classification, can serve as a clinically useful framework for understanding the involvement of the PT in Mid-AT and guiding appropriate surgical intervention. Particular emphasis is placed on whether certain types are more likely to contribute to pathology and whether their identification should influence therapeutic decision-making.

## 2. Anatomy and Variability of the Plantaris Tendon

### 2.1. Methodological Note

This narrative review was conducted using a structured literature search strategy to ensure comprehensive and clinically relevant coverage of anatomical and radiological aspects of the plantaris tendon (PT) and its role in midportion Achilles tendinopathy (Mid-AT). The literature search was performed across PubMed, MEDLINE, and Scopus, focusing on studies published between 1900 and May 2025. The inclusion of both contemporary clinical studies and classical anatomical literature was intentional, to reflect the historical evolution of plantaris tendon understanding.

The search terms used included the following: “plantaris tendon” OR “plantaris muscle” AND “Achilles tendinopathy” OR “Achilles tendon” AND “anatomical variation” OR “classification” OR “MRI” OR “ultrasound”. Only articles written in English were included. No restrictions were applied regarding study design or country of origin.

A total of 38 publications were included in the review. These comprised cadaveric anatomical studies, imaging-based investigations (MRI, ultrasound, elastography), surgical case series, clinical reports, and selected developmental references. Studies were screened based on:

- Relevance to adult human anatomy;
- Clinical implications in Mid-AT;
- Description of plantaris–Achilles anatomical relationships;
- Utility in establishing or validating classification systems.

To minimize selection bias, the literature was reviewed by multiple co-authors independently. The classification proposed by Olewnik et al. was used as the main reference framework, but alternative systems were also discussed where appropriate to ensure objectivity.

### 2.2. Location, Course, and Relation to the Achilles Tendon

The plantaris muscle typically originates from the lateral supracondylar line of the femur and the oblique popliteal ligament. It gives rise to a long, slender tendon that courses distally between the gastrocnemius and soleus muscles before descending along the medial side of the AT. Its distal insertion is most commonly on the medial aspect of the calcaneus, although significant variation exists [3,4]. Due to its proximity to the AT, the PT can lie directly adjacent to or even interdigitate with the Achilles, which may lead to pathological friction or compression, particularly in the context of Mid-AT [12,18].

### 2.3. Histological Structure and Biomechanical Properties

Histologically, the plantaris tendon is composed of dense, regularly arranged collagen fibers similar to those of other load-bearing tendons. However, the collagen fiber orientation and interfascicular matrix have been shown to differ from that of the AT, suggesting different loading capacities and biological responses [14]. Some studies have identified cholinergic nerve fibers within the PT, suggesting a potential sensory or modulatory role in tendon pain [14]. From a biomechanical perspective, the PT exhibits less tensile strength than the AT, and its anatomical course, particularly when medially displaced or twisted, may predispose it to shear-related stress against the AT [13,19,20].

### 2.4. Review of Morphological Variations in Adult Populations

Morphological variability of the PT is well documented. Daseler and Anson [21] first reported a plantaris presence in 89 percent of cadaveric specimens, while more recent studies have confirmed frequent variations in origin, course, and insertion. Olewnik et al. [4] introduced a classification system encompassing multiple types based on the relationship between the PT and the AT, ranging from completely separate tendons to those tightly adherent or partially fused. Rare cases of dual insertions, three-headed or four-headed origins, or even complete absence have also been described [22,23,24,25,26]. This high degree of variability has significant implications for both diagnosis and surgical intervention, particularly in patients with refractory Mid-AT.

## 3. Classification of the Plantaris Tendon According to Olewnik et al. [21] (Types I–VI)

The morphological variability of the PT was initially classified by Olewnik et al. [4] based on anatomical dissection of 50 lower limbs. In this study, five distinct types of insertion were identified, each characterized by its location on or around the calcaneal tuberosity or deep fascia, and its spatial relationship to the AT:

- Type I: Fan-shaped insertion on the medial side of the calcaneal tuberosity.
- Type II: Insertion close to the AT, typically within a shared paratenon.
- Type III: Insertion anterior to the AT.
- Type IV: Insertion into the deep crural fascia, with no contact with the calcaneus.
- Type V: Wide insertion encircling posterior and medial surfaces of the AT.

Subsequently, in a larger anatomical study involving 130 lower limbs, Olewnik et al. [3] extended the previous classification by adding a sixth type (Type VI), describing a rare insertion of the PT into the flexor retinaculum of the tarsal canal, which may be relevant not only to Achilles tendinopathy but also to tibialis posterior pathology. This second classification builds directly upon the 2017 scheme, retaining the same five types and formally introducing:

- Type VI: Insertion into the tarsal canal flexor retinaculum, located anterior to the AT and calcaneus.

Both classifications also describe two main variants of the tendon’s course:

- Variant A: Tendon runs along the medial side of the AT.
- Variant B: Tendon crosses anteriorly in front of the AT, seen most commonly in Types III, IV, and VI.

The progression from five to six types reflects enhanced anatomical resolution and highlights the clinical significance of subtle variations, especially in patients with refractory Achilles midportion tendinopathy. Table 1 presents the detailed classification of the plantaris tendon according to Olewnik et al., including six morphological types based on insertion site, anatomical course, prevalence, and the potential risk of mechanical conflict with the Achilles tendon.

### 3.1. Exclusion of Fetal Classifications and Other Non-Clinical Models

Several studies have proposed classifications of the plantaris tendon based on fetal dissections. Notably, Waśniewska et al. [27] examined morphological variability in human fetuses, identifying early developmental variants in the origin and insertion of the plantaris muscle. While these findings enrich embryological understanding, they do not reflect mature tendon structure or load-adaptive properties observed postnatally. As such, fetal classifications lack direct applicability to adult tendinopathy or surgical intervention planning.

Furthermore, classifications derived from isolated anatomical cases without clinical correlation—such as those reported by Nayak et al. [28] and Simpson et al. [29] were excluded. These systems, often focused on tendon graft availability or rare morphologies, do not offer insight into dynamic tendon–Achilles interactions, nor have they been validated against clinical imaging or treatment outcomes [11,12].

### 3.2. Comparison with the van Sterkenburg Classification

The observational anatomical study by van Sterkenburg et al. [30] described nine distinct insertion patterns of the PT, including medial, posteromedial, anteromedial, and anterior attachments to the calcaneus, as well as insertions into the deep fascia. Although this classification offers high descriptive granularity, it lacks a systematic framework and does not incorporate tendon course variants or relate findings to tendinopathy.

Importantly, the study did not assess relationships with the AT in a manner conducive to clinical decision-making, nor was it connected with therapeutic algorithms or imaging protocols. For these reasons, the van Sterkenburg classification was not adopted in the present review. In contrast, the system developed by Olewnik et al. [3] allows for anatomical typing that can be visualized preoperatively and addressed during surgical exploration.

One of the authors of this review (Ł.O.) is also the primary developer of the PT classification that forms the conceptual foundation of the present work. To ensure scientific integrity and minimize the risk of author bias, the manuscript was prepared through a structured and collaborative process involving co-authors with diverse backgrounds in clinical anatomy, orthopaedic surgery, radiology, and musculoskeletal research. The selection and interpretation of relevant studies including cadaveric, imaging-based, and clinical sources were conducted independently by multiple contributors. Critical appraisal of alternative classification systems, such as those proposed by van Sterkenburg et al. [31] and Nayak et al. [15], was incorporated to contextualize the strengths and limitations of the Olewnik classification.

The decision to use the Olewnik system as the central framework was based on its demonstrated reproducibility, integration of both course variants and insertion types, and its established utility in correlating anatomical findings with diagnostic imaging and surgical outcomes. In contrast to earlier descriptive schemes, this classification has been validated across multiple methodological platforms and provides stratified, type-specific guidance for both conservative and surgical management of midportion Achilles tendinopathy.

### 3.3. Developmental Origins of Morphological Variants

The morphological variability of the PT observed in adult anatomy likely stems from developmental processes occurring during early myogenesis. The PM originates from the posterior condensation of the paraxial mesoderm in the lower limb bud, alongside the gastrocnemius and soleus muscles, as part of the superficial flexor group of the posterior compartment [4,5]. During normal embryogenesis, these muscle primordia undergo differentiation, migration, and fusion events that establish the definitive muscle-tendon units.

Aberrations in this process, such as incomplete regression, asymmetric migration, or altered fusion with adjacent structures (e.g., crural fascia or paratenon of the Achilles tendon), are believed to account for the range of adult anatomical variants [32,33]. For instance, Type IV insertions, which terminate in the deep fascia, may represent incomplete distal migration. Type V, with broad contact along the Achilles tendon, could be the result of excessive fusion or lack of fascial separation. The rare Type VI variant, inserting into the flexor retinaculum, may be associated with a developmental trajectory closely aligned with the tibialis posterior compartment, potentially due to medial misrouting during distal extension of the myotome.

Human fetal dissections have demonstrated that the plantaris tendon displays significant morphological heterogeneity already by the second trimester, including accessory heads, aberrant insertions, and even complete absence [32,34]. These findings support the notion that the six adult types described in the Olewnik classification may reflect stabilized developmental variants arising from subtle changes in myotomal patterning, segmental innervation, or tendon morphogenesis.

Understanding these embryological underpinnings is not only important from a theoretical standpoint, but also offers a biological rationale for the observed structural variations that contribute to differential clinical presentations and treatment outcomes in midportion Achilles tendinopathy.

## 4. Midportion Achilles Tendinopathy (Mid-AT)

### 4.1. Pathophysiology: Mechanical Microtrauma and Collagen Degeneration

The Mid-AT is a chronic, non-inflammatory condition characterized by pain, swelling, and impaired load-bearing function located 2–7 cm proximal to the tendon’s calcaneal insertion. The underlying pathophysiology is now understood to involve a degenerative rather than inflammatory process, in which repetitive mechanical loading leads to microtrauma, disorganization of collagen fibers, and a failed healing response [8,9].

Histological findings in affected tendons typically include collagen fiber disarray, increased tenocyte activity, and the accumulation of glycosaminoglycans, but without inflammatory cell infiltration [11,12]. These changes are associated with the replacement of strong, well-aligned Type I collagen by structurally inferior Type III collagen, resulting in diminished tensile strength and viscoelasticity of the tendon. Additionally, neovascularization and sensory nerve ingrowth into the degenerated region are thought to be key contributors to pain perception in chronic tendinopathy [14,35].

Biomechanically, the condition is exacerbated by eccentric tensile overload and localized compressive forces, especially in individuals participating in high-impact activities such as running or jumping [10,11]. These forces may be aggravated by adjacent anatomical structures, such as the plantaris tendon, which has been implicated as a mechanical stressor due to its course and potential compression against the medial AT in select anatomical variants [12,36].

In summary, Mid-AT represents a continuum of failed tendon repair, progressing from asymptomatic degeneration to persistent pain and dysfunction, often resistant to conservative treatment and requiring surgical consideration.

### 4.2. The Role of Plantaris–Achilles Friction

An increasing body of evidence supports the hypothesis that friction or mechanical interaction between the PT and AT may contribute significantly to the pathogenesis of midportion Achilles tendinopathy (Mid-AT), especially in cases presenting with localized medial-sided pain. This concept has gained attention following both anatomical and clinical studies demonstrating the close proximity and variable relationship between the two tendons.

The PT typically courses medial or anteromedial to the AT, but in several anatomical variants, it lies in direct contact or even shares a common paratenon with the AT [3,4]. These configurations may create zones of friction, particularly during eccentric loading of the triceps surae complex, resulting in shearing forces, local inflammation of the paratenon, or mechanical irritation of the AT itself [12,19].

Histological evidence supports this mechanical conflict model. In their analysis of resected plantaris tendons in patients with chronic Mid-AT, Spang et al. [14] described tendinosis-like changes within the plantaris itself, along with altered extracellular matrix composition and cholinergic innervation. These features are consistent with a structure that is not merely vestigial, but rather functionally implicated in the disease process.

Clinical observations align with these findings. In a large surgical case series, Alfredson et al. [12] reported that nearly one-fifth of patients with chronic Mid-AT who failed conservative treatment had a macroscopically normal AT but an abnormal, thickened, or malpositioned PT. Notably, removal of the PT alone led to symptom resolution in many of these cases [16,36].

Imaging modalities such as ultrasound and high-resolution MRI are increasingly used to evaluate plantaris–Achilles interactions. However, due to the slender and variable course of the PT, diagnosis remains challenging, and surgical exploration often reveals findings not captured on preoperative imaging [17,19].

Thus, anatomical variants of the PT should be regarded as potential extrinsic contributors to medial-sided Achilles pain, and their identification is crucial in treatment-resistant cases. The Olewnik classification provides a useful framework for anticipating these interactions and guiding therapeutic strategy.

### 4.3. Clinical Presentation and Physical Examination

The Mid-AT most commonly presents with gradual-onset pain localized 2–7 cm proximal to the AT insertion. The pain is typically worse during or after activity and improves with rest. Patients often report morning stiffness, discomfort while ascending stairs, or pain during walking and running [8,10].

On physical examination, the hallmark finding is localized tenderness over the midportion of the tendon, usually on the medial side, especially in cases involving plantaris-related friction. Swelling or fusiform thickening of the tendon may be observed, and in chronic cases, a nodular or irregular texture can be palpated. Pain is usually reproducible with single-leg heel raises or hopping [9,11].

Clinical suspicion should be heightened in patients with medial-sided pain that is disproportionate to imaging findings involving the AT itself. This may suggest an underlying plantaris conflict [12,14].

### 4.4. The Role of Imaging (Ultrasound, MRI, Elastography)

Imaging plays a critical role in confirming the diagnosis of Mid-AT, identifying plantaris involvement, and planning surgical intervention. Ultrasound (US) is often the first-line tool due to its accessibility, dynamic capability, and high resolution. It can detect tendon thickening, hypoechoic areas, neovascularization, and alterations in tendon echotexture [17,35].

Color and power Doppler imaging are valuable for visualizing neovascularization, which has been associated with pain in tendinopathy. However, the thin and variable course of the PT may limit its detectability on routine ultrasound, requiring high-resolution probes and meticulous scanning techniques [17,19].

Magnetic Resonance Imaging (MRI) offers excellent soft tissue contrast and may better delineate the relationship between the PT and AT, particularly in Types II, V, and VI of the Olewnik classification. MRI can also assess surrounding edema, fluid collections, or retrocalcaneal bursitis when present [30,31].

Ultrasound elastography, a newer technique, provides quantitative assessment of tendon stiffness and may help in early detection of degenerative changes before morphological alterations are apparent on conventional US or MRI [17,34].

Thus, a multimodal imaging approach combining grayscale US, Doppler, and MRI—augmented by elastography when available—offers the best diagnostic accuracy and preoperative planning capability in patients with recalcitrant Mid-AT.

## 5. Association of Plantaris Tendon Types with Mid-AT

### 5.1. Clinical and Cadaveric Evidence Linking Plantaris Tendon Types to Mid-AT

Over the past decade, growing interest in the role of the plantaris tendon in Mid-AT has prompted several cadaveric and clinical investigations aimed at establishing correlations between specific anatomical variants and symptomatic tendinopathy. These studies consistently indicate that certain insertion types and tendon courses, as defined in the Olewnik classification, exhibit a greater potential for mechanical conflict with the AT.

In a foundational anatomical study, Olewnik et al. [4] examined 50 lower limbs and identified five primary insertion types, later expanded to six in a larger series of 130 limbs [3]. They reported that Types II, III, V, and VI are most likely to be implicated in Mid-AT due to either direct contact with the AT, shared paratenon, or encircling insertion. Variant A, which places the tendon medially in close proximity to the AT, was observed in over 80% of cases and includes Types I, II, and V whereas Variant B, associated with Types III, IV, and VI, demonstrates anterior or fascia-based insertion, often producing atypical symptomatology.

These cadaveric findings are strongly supported by clinical data. In a large case series, Alfredson et al. [12] documented that approximately 20% of patients undergoing surgical treatment for Mid-AT had a normal AT but a hypertrophic or pathologically positioned plantaris tendon, most often corresponding to Type II or V. Resection of the plantaris tendon in such cases resulted in substantial symptom relief, reinforcing the causative biomechanical role of these types.

Similarly, Masci et al. [36] reported improvements in pain and tendon structure after targeted AT scraping and PT excision, with ultrasound and intraoperative findings indicating plantaris–Achilles fusion or impingement in the majority of cases. Notably, MRI and high-resolution ultrasound were able to detect plantaris variants correlating to Types II and VI in a subset of patients with persistent medial pain and previously inconclusive imaging [17,19].

Further anatomical support comes from Spang et al. [14], who described degenerative changes in excised PT, including neovascularization and cholinergic innervation, indicating an active role in the pain mechanism. These histological findings align with imaging and dissection-based observations that anatomical conflict is not merely spatial but pathophysiological.

Taken together, these studies form a consistent body of evidence showing that PT Types II, V, and VI, especially in conjunction with Variant A are clinically significant in the pathogenesis and persistence of Mid-AT, and their recognition is essential for individualized surgical planning.

### 5.2. Conflictogenic Risk of Individual Plantaris Tendon Types

The anatomical relationship between the plantaris and Achilles tendons is highly variable, and this variability directly influences the likelihood of mechanical conflict. The classification proposed by Olewnik et al. [4], based on 50 cadaveric lower limbs, and expanded in Olewnik et al. [3] to 130 limbs, enables a detailed risk assessment based on tendon insertion type and trajectory.

Type I (fan-shaped insertion on the medial calcaneus) and Type V (encircling the AT) are associated with the highest conflictogenic potential. These types present extensive contact surfaces and are frequently involved in friction, dynamic compression, and paratenon irritation, especially when accompanied by Variant A, which places the tendon along the medial side of the AT [3,4].

Types II and III are considered to have moderate risk. Type II lies adjacent to the AT and often shares a common paratenon, a configuration repeatedly visualized in high-resolution ultrasound and MRI in symptomatic patients [17]. Type III, inserting anterior to the AT, has a more oblique and superficial course and may generate shearing stress during movement [19].

Type IV, which inserts into the deep crural fascia, has no structural connection with the AT and is regarded as clinically irrelevant in terms of Mid-AT [3,4].

Type VI inserts into the flexor retinaculum, near the tarsal canal. Though not in direct contact with the AT, it has been hypothesized to cause tibial nerve compression, mimicking tarsal tunnel syndrome (TTS)**.** This has been described in both fetal and adult anatomical studies and may be especially relevant in atypical clinical presentations [3,32].

### 5.3. Clinical Recommendations Based on Plantaris Tendon Type

Accurate identification of the plantaris tendon type is essential in patients with Mid-AT who fail to improve with conservative management. The anatomical insights provided by the Olewnik classification help stratify patients for individualized treatment, particularly in surgical planning.

For Types I and V, which show the strongest anatomical and clinical association with Mid-AT, early surgical intervention is often warranted. Alfredson et al. [15] reported that nearly 20% of patients undergoing surgery had a macroscopically normal AT but an abnormal PT—most commonly of Type I or V. Removal of the PT alone led to marked pain relief, underscoring its pathophysiological role. Imaging with US or MRI should be used to confirm PT course and contact zones preoperatively [17].

Types II and III may initially be managed conservatively, though persistent symptoms—particularly medial-sided tenderness in Type II and anterior Achilles pain in Type III—may justify surgical removal. In these cases, preoperative ultrasound and intraoperative inspection are useful in confirming mechanical conflict [17,19].

Type IV poses no meaningful mechanical risk and should not be addressed surgically. Its identification should direct clinicians to evaluate other causes of symptoms [3].

Type VI should prompt consideration of neurological assessment, particularly in patients presenting with posteromedial ankle pain, paresthesia, or TTS-like symptoms. High-resolution MRI is recommended. In confirmed cases of compression, selective plantaris excision or neurolysis may be appropriate [3,32].

These evidence-based recommendations support an anatomy-guided approach to treatment, improving diagnostic precision and avoiding unnecessary interventions.

A summary of conflictogenic risk and recommended management by type is presented in Table 2.

## 6. How to Identify Plantaris–Achilles Conflict

Accurately diagnosing plantaris–Achilles conflict in patients with Mid-AT can be challenging due to the overlap in clinical symptoms with other pathologies and the subtlety of anatomical variations. However, growing anatomical and imaging-based evidence now provides clear criteria to guide recognition of this condition.

### 6.1. Clinical Clues

Patients typically present with medial-sided Achilles pain localized between 2 and 7 cm proximal to the calcaneal insertion. The pain is often described as sharp or localized rather than diffuse, and is reproducible with palpation over the medial tendon border [12]. In cases involving plantaris conflict, patients may report worsening of pain with eccentric loading, especially when conservative treatment has failed.

Clinically, suspicion should be raised in the following contexts:

- Medial pain with a structurally normal AT on imaging;
- No response to traditional tendinopathy treatments (eccentric training, shockwave therapy);
- Pain reproduction during passive dorsiflexion combined with inversion;
- Palpable cord-like structure medial to the AT [4].

### 6.2. Ultrasound Examination

High-resolution ultrasound (US) is a first-line imaging modality due to its dynamic capability. The PT may appear as a thin, hyperechoic band medial or anterior to the AT. However, due to its small size and variable course, visualization can be difficult, requiring careful scanning in both axial and longitudinal planes [17,19].

Color Doppler ultrasound may reveal hypervascularity in the plantaris–Achilles interface, particularly in cases with pain during tendon compression or motion. A shared paratenon or intratendinous fusion with the AT may be visible in Type II or V [36].

### 6.3. Magnetic Resonance Imaging (MRI)

MRI is especially useful when ultrasound is inconclusive or to assess deep tendon relationships. Sagittal and axial T1- and T2-weighted sequences may identify:

- Anatomical proximity or adhesion between plantaris and AT
- Peritendinous edema or focal thickening at the medial Achilles border
- Course and insertion of the PT corresponding to Type II, V or VI [30,31].

MRI is also critical in detecting Type VI, which may mimic tarsal tunnel syndrome due to its posteromedial trajectory toward the flexor retinaculum.

### 6.4. Elastography

Ultrasound elastography offers a quantitative assessment of tendon stiffness and has shown promise in detecting early-stage tendinopathy, especially when standard grayscale findings are subtle [17,36]. While still limited in availability, elastography may help differentiate plantaris-induced stiffness zones from focal Achilles degeneration.

### 6.5. Role of Classification in MRI and Ultrasound Assessment

The use of a standardized anatomical classification of the PT, such as the Olewnik system, significantly enhances the diagnostic value of imaging modalities including US and MRI. This classification not only facilitates consistent identification of tendon variants but also improves the clinician’s ability to correlate anatomical findings with clinical symptoms in patients with Mid-AT.

In US evaluation, recognition of the type of plantaris insertion and its spatial course relative to the AT can guide the examiner to areas of likely friction or impingement. For example, Type II insertions often demonstrate a thin hyperechoic tendon lying directly adjacent to the AT, occasionally with visible blending of paratenon layers. Types I and V insertions may appear as broad echogenic bands encircling or compressing the medial or posterior aspect of the AT [17,19]. Dynamic ultrasound assessment during passive dorsiflexion and inversion may enhance visualization of mechanical interaction, particularly in patients with variant A PT courses.

MRI also benefits from classification-based interpretation. In axial and sagittal views, the location and trajectory of the PT can be mapped in relation to the AT, allowing for the identification of fusion zones, proximity patterns, or aberrant insertions. Types I and V typically correlate with visible structural overlap, thickened paratenon, or peritendinous edema. Type VI insertions, which are easily overlooked, may be traced to the flexor retinaculum or adjacent neurovascular structures, providing important diagnostic cues in patients with atypical or neuropathic symptoms [3,30].

Importantly, the classification system allows radiologists and clinicians to move beyond binary descriptors such as “present” or “absent” and instead report specific morphological subtypes, which can be documented and monitored across imaging sessions or postoperatively. This is particularly relevant in longitudinal studies or in surgical planning, where detailed anatomical documentation influences the decision to perform PT excision, scraping, or decompression.

Thus, incorporating the Olewnik classification into MRI and US interpretation enables a more nuanced and clinically actionable assessment of plantaris–Achilles interactions, helping to bridge the gap between anatomical variation and symptom origin.

### 6.6. Importance in Surgical Qualification

Accurate identification of plantaris tendon morphology plays a central role in qualifying patients with Mid-AT for surgical intervention. Increasing clinical and anatomical evidence suggests that failure to respond to conservative treatment may, in a substantial subset of cases, be attributed not to intrinsic AT degeneration but to mechanical conflict with a structurally variant PT.

Anatomical types defined in the Olewnik classification provide a framework for stratifying risk and determining which patients may benefit from surgical excision of the PT. For example, Types I and V, which show broad or encircling insertions and direct contact with the AT, are consistently associated with intraoperative findings of paratenon adhesion, tendon thickening, and friction zones. These types are considered strong surgical indications, especially in patients with persistent medial-sided pain despite structured rehabilitation [3,15].

In contrast, Type IV, which inserts into the deep fascia without contacting the AT, generally does not require intervention, and its identification may prevent unnecessary surgery. Type VI, though not directly implicated in Achilles tendinopathy, may justify surgical management in the context of tibial nerve compression or tarsal tunnel syndrome-like symptoms [32].

Preoperative imaging (ultrasound, MRI) plays a crucial role in this decision-making process. When imaging confirms a conflictogenic variant (e.g., Type II or V with shared paratenon or medial tethering), and symptoms are reproducible with mechanical stress, surgical removal of the PT often leads to symptom resolution [17,36]. Moreover, the presence of a structurally normal AT in such patients supports the view that the plantaris may be the primary pain generator.

Ultimately, the integration of classification-guided imaging with physical examination and clinical history enables a more precise and individualized approach to surgical qualification. It allows for selective intervention, avoiding overtreatment and reducing postoperative failure by ensuring that the target of surgery corresponds anatomically and symptomatically to the source of pathology.

## 7. Orthopaedic Management According to Plantaris Tendon Type

### 7.1. Conservative Treatment (Eccentric Exercises, HSR, ESWT, PRP)

Conservative management remains the first-line treatment in patients with Mid-AT, and its effectiveness is well-documented, particularly in early or moderate cases. The choice of modality and its duration, however, should be guided by the anatomical subtype of the PT and the likelihood of mechanical conflict.

Eccentric loading programs have long been considered the gold standard in tendinopathy treatment. The Alfredson protocol, based on repetitive eccentric heel drops, has shown high success rates in chronic cases, improving tendon structure and reducing pain over 12-week periods [12]. This form of loading may promote collagen realignment, stimulate tenocyte activity, and reduce neovascularization.

More recently, heavy slow resistance (HSR) protocols have emerged as effective alternatives or adjuncts to eccentric training. HSR protocols employ high-load, low-velocity exercises performed in a controlled manner, often yielding similar or superior results in tendon remodeling and pain reduction [11]. These programs may be particularly suitable in athletes or patients intolerant to high-repetition eccentric protocols.

In addition to loading strategies, adjunctive therapies such as extracorporeal shock wave therapy (ESWT) and platelet-rich plasma (PRP) injections are commonly used. ESWT has demonstrated variable efficacy but may provide benefit in recalcitrant cases through promotion of neovascular regression, analgesia, and stimulation of tissue healing [33]. PRP injections, although controversial, are employed to introduce autologous growth factors into the degenerative tendon and have shown mixed outcomes across trials. Their utility may depend on precise localization and ultrasound guidance, especially in patients with detectable plantaris–Achilles conflict [17].

Importantly, PT variants with high conflictogenic potential, such as Types I, II, and V, may respond poorly to conservative strategies alone. In such cases, even well-executed eccentric or HSR regimens may fail to resolve symptoms due to persistent mechanical interference. Recognition of such anatomical patterns early in the course of treatment may help avoid unnecessary prolongation of nonoperative care.

Patients with Type IV, or with no imaging signs of PT involvement, are more likely to benefit from prolonged conservative therapy. For Type VI, particularly when neurovascular symptoms are absent, conservative treatment may also be pursued before considering surgery.

Thus, an anatomy-informed approach to conservative management not only supports individualized rehabilitation strategies but may also help identify patients who are less likely to benefit from prolonged nonoperative care.

### 7.2. Surgical Treatment Stratified by Plantaris Tendon Type

Surgical intervention for Mid-AT should be tailored according to the anatomical type of the PT. Below is a structured overview of recommended procedures based on the Olewnik classification, incorporating both operative technique and clinical considerations—Table 3.

Types I and V insertions are most frequently associated with persistent medial-sided pain, structural impingement, and intraoperative findings of paratenon adhesion. These types often require open access due to their wide insertion surface and proximity to the AT. Types II and III can typically be addressed endoscopically, especially when imaging reveals a discrete tendon with limited overlap.

Type IV does not interact with the AT and should not be considered a surgical target. Type VI requires careful preoperative evaluation for potential tibial nerve compression, and surgical management may involve both PT excision and neurolysis.

This stratified approach aims to optimize surgical outcomes by aligning the operative technique with anatomical risk and symptomatology.

## 8. Clinical Outcomes of Surgical Treatment for Midportion Achilles Tendinopathy

### 8.1. Review of Outcomes: Effectiveness of Plantaris Tendon Resection

Several studies consistently demonstrate that surgical removal of the PT leads to substantial pain relief and functional improvement in patients with midportion Mid-AT, particularly when conservative treatment has failed. Success rates range between 80% and 90% across published case series.

Alfredson et al. [15], in a large WALANT surgical cohort, reported that approximately one-fifth of tendons appeared macroscopically normal intraoperatively, yet showed pathological PT. These patients experienced significant symptom relief following isolated PT excision (Tendinopathic plantaris but normal Achilles tendon found in about one-fifth of patients not responding to conservative Achilles management).

Masci et al. [17] demonstrated that plantaris excision combined with AT scraping led to structural tendon improvement confirmed by ultrasound tissue characterization at 24-month follow-up (Achilles scraping and plantaris tendon removal improves pain and tendon structure in patients with mid-portion Achilles tendinopathy).

Histopathological studies, including those by Spang et al. [14], confirmed tendinosis-like changes in excised plantaris tendons, including increased vascularity and presence of a non-neuronal cholinergic system, supporting the notion that the plantaris is an active pain generator (The plantaris tendon in association with mid-portion Achilles tendinosis: tendinosis-like morphological features and presence of a non-neuronal cholinergic system).

### 8.2. Comparison of Surgical Techniques: Open vs. Endoscopic Resection

Open resection is traditionally used when the PT is broad, adherent, or fused with the AT, as in Types I and V. It offers clear visualization and the ability to perform concurrent Achilles debridement, but carries a higher risk of wound complications and longer recovery [3].

Endoscopic resection, on the other hand, is minimally invasive and typically used in Types II and III, where the tendon lies free and superficial. Smith et al. [19] described sonographic evidence of differential tendon motion, which correlated with surgical findings in endoscopic cases. While endoscopy provides faster rehabilitation, it demands higher precision and preoperative imaging accuracy.

Although no large randomized controlled trials compare the two techniques, observational evidence suggests that both yield favorable outcomes when anatomically matched [17,36].

### 8.3. Complications and Their Association with Tendon Type

Complications after plantaris excision are rare but include persistent medial pain, incomplete symptom resolution, and occasionally sural nerve irritation. These issues are most frequently observed in cases where the PT is partially fused with the AT (Type II) or where incomplete resection occurs [31,36].

Type V, with its broad enveloping insertion, poses a higher technical challenge, especially in endoscopic approaches, increasing the risk of residual conflict. Conversely, unnecessary resection in Type IV—which has no anatomical interaction with the AT—may result in avoidable morbidity.

In rare Type VI cases, failure to decompress the tibial nerve may result in persistent neuropathic symptoms despite plantaris excision. This underscores the need for thorough anatomical and neurovascular assessment, especially in atypical or refractory presentations [3,32].

### 8.4. Importance of Precise Matching Between Anatomical Type and Surgical Technique

Effective surgical management of Mid-AT depends not only on the decision to excise the PT, but also on a detailed understanding of its anatomical variant. Increasing anatomical and clinical evidence supports the principle that precise alignment between the PT type and the chosen surgical approach improves patient outcomes, minimizes complications, and reduces the likelihood of symptom recurrence.

Types I and V are characterized by broad, adherent insertions along the medial or posterior AT, often associated with paratenon fusion or circumferential contact. These configurations frequently require open surgical exposure to allow for adequate visualization and complete resection. Open techniques also enable concurrent debridement of the Achilles paratenon or scraping of degenerative tissue, which is frequently present in these conflictogenic types [3,15].

Types II and III, on the other hand, generally present with discrete, slender tendons located superficially or anteriorly relative to the AT. These are often accessible via minimally invasive approaches, such as endoscopic resection. When preoperative imaging confirms clear separation from the AT and no fusion planes, endoscopic removal may reduce surgical morbidity and promote faster rehabilitation. However, failure to adapt the technique to the tendon’s trajectory may lead to incomplete excision, persistent symptoms, or iatrogenic injury [17,19].

Type VI presents a unique challenge due to its atypical course toward the flexor retinaculum and potential involvement of the tibial nerve. In such cases, a tailored approach including nerve decompression should be considered, and the PT should not be addressed in isolation. Failure to recognize the neurovascular implications of this type may result in suboptimal outcomes [3,32].

Applying a uniform surgical technique without anatomical stratification may result in unnecessary procedures (as in Type IV), incomplete removal (as in Type V), or failure to address nerve-related symptoms (as in Type VI). Therefore, classification-guided planning ensures that the choice between open or endoscopic access, the extent of excision, and adjunctive procedures (e.g., neurolysis or Achilles scraping) are fully individualized.

This anatomical-surgical alignment reflects a broader trend toward precision orthopaedic treatment and supports the integration of preoperative imaging, intraoperative assessment, and evidence-based classification systems into clinical practice.

## 9. Significance of the Olewnik Classification in Orthopaedic Practice

### 9.1. Assistance in Treatment Planning

The anatomical classification developed by Olewnik et al. provides a structured and clinically applicable framework for managing Mid-AT, particularly when PT involvement is suspected. By organizing the six known anatomical variants based on their spatial relation to the AT and their morphological features (e.g., insertion site, breadth of attachment, course), this classification supports a precise, anatomy-based therapeutic decision-making process [3]. It enables clinicians to identify high-conflict variants—such as Types I and V—where the PT inserts closely and broadly onto the medial AT, often causing friction, tethering, or compression, and where open surgical intervention combined with Achilles debridement may be necessary [15]. Meanwhile, Types II and III, which course parallel but maintain less intimate contact, are typically amenable to minimally invasive endoscopic excision with reduced morbidity [14,18,30].

### 9.2. Avoidance of Overdiagnosis or Unnecessary Surgery

A major benefit of the classification lies in its potential to reduce overtreatment and unnecessary surgical exploration. Not all patients with Mid-AT benefit from plantaris excision—particularly those with isolated Achilles degeneration. The identification of Type IV, where the PT is completely independent from the AT and lacks frictional potential, helps delineate patients who are best managed conservatively [15,17]. Furthermore, Type VI represents a rare variant with a unique medial path and tibial nerve compression potential. Recognizing this configuration is crucial, as it can mimic tarsal tunnel syndrome (TTS), and incorrect diagnosis may result in ineffective Achilles-focused surgery. In such cases, targeted decompression of the tibial nerve combined with tendon resection yields superior outcomes [32].

### 9.3. Educational and Teaching Potential

In orthopaedic residency programs and postgraduate sports medicine training, the Olewnik classification can be used as an educational scaffold for teaching musculoskeletal anatomy and pathology. Its incorporation into imaging interpretation curricula has already improved recognition of pathological variants on MRI and high-resolution ultrasound [15,19]. For anatomists and medical educators, the classification underscores the dynamic variability of human tendon architecture and its relevance to clinical syndromes. As surgical management becomes increasingly personalized, the ability to predict symptom etiology based on morphology has strong pedagogical value.

### 9.4. Proposal of a Decision-Making Algorithm

The classification allows for the construction of a reproducible clinical algorithm that guides both diagnosis and intervention—Table 4.

Such an algorithm encourages standardized clinical reasoning and interdisciplinary dialogue between surgeons, radiologists, and physical therapists. Importantly, it provides a basis for prospective outcome stratification and comparative studies between PT types [3,14,15,18].

## 10. Future Research Directions

### 10.1. Need for Prospective Studies with Pre- and Post-Treatment MRI

Despite increasing recognition of the plantaris tendon’s role in Mid-AT, most evidence remains retrospective and lacks robust imaging-based follow-up. Future studies should employ standardized MRI protocols before and after conservative or surgical treatment to assess morphological changes, tendon signal alterations, and decompression efficacy. Such prospective trials would validate anatomical classifications against clinical outcomes and improve selection criteria for operative intervention [15,17].

### 10.2. Potential Integration of the Classification with Bioinformatics Tools

The Olewnik classification offers a structural basis for computational modeling of PT interactions. Integration with AI-driven image recognition systems, 3D tendon reconstruction, and machine learning algorithms could enable semi-automated diagnosis, conflict prediction, and personalized surgical planning. Development of digital platforms incorporating real-time ultrasonography data with classification-matching software may streamline workflows and reduce diagnostic variability across institutions [3,32].

### 10.3. Application in Targeted Therapies—Plantaris Tendon as an Autologous Graft Source

An intriguing future direction involves repurposing the PT, particularly in non-conflict types (e.g., Type IV), as a potential autologous graft for reconstructive procedures. Its accessibility, minimal donor site morbidity, and histological similarity to other tendon tissues make it a promising candidate in ligament repair or augmentation strategies. Integrating anatomical classification could refine graft selection criteria and optimize surgical outcomes in both orthopaedic and sports medicine contexts [19].

## 11. Conclusions

The classification developed by Olewnik et al. enables a precise match between the treatment strategy and the specific morphology of the plantaris tendon. Its highest clinical utility is demonstrated in Types I and V, where anatomical conflict is most pronounced and surgical intervention is frequently warranted. As a whole, this classification represents an important tool in the management of patients with chronic midportion Achilles tendinopathy (Mid-AT), offering a structured framework that can enhance the outcomes of surgical therapy and guide personalized treatment approaches.

## Figures and Tables

**Table 1:** Classification of the plantaris tendon according to Olewnik et al.

Type	Insertion Site	Course Variant	Prevalence (%)	Risk of Achilles Conflict
I	Fan-shaped on medial calcaneal tuberosity	A	44	Low
II	Medial calcaneal tuberosity, shared paratenon with Achilles	A	22.4	High (shared sheath)
III	Anterior to Achilles on calcaneus	B	6.9	Moderate (anterior shear)
IV	Deep crural fascia (not on calcaneus)	B	3.4	Low
V	Encircling posterior and medial Achilles surfaces	A	18.1	High (encircling contact)
VI	Flexor retinaculum near tarsal canal	B	5.2	High (close anterior relation)

**Table 2:** Conflictogenic risk and clinical recommendations by plantaris tendon type.

Plantaris Tendon Type	Conflictogenic Potential	Pathophysiological Basis	Clinical Recommendation
Type I	High	Fan-shaped medial insertion with broad contact area; friction risk increased with medial (Variant A) course	Recommend early surgical excision if symptoms persist; evaluate with high-resolution ultrasound/MRI
Type II	Moderate	Insertion close to Achilles tendon, often within a shared paratenon	Consider surgical removal in persistent medial-sided pain; visible on MRI or ultrasound
Type III	Moderate	Anterior to Achilles; shearing risk due to crossing trajectory (Variant B)	Surgery considered if anterior symptoms match imaging; assess intraoperatively
Type IV	Low	Insertion into deep crural fascia; no anatomical contact with Achilles	Exclude from surgical consideration; focus on other etiologies
Type V	High	Encircling posterior-medial Achilles; strong anatomical association with Mid-AT	Strong indication for plantaris excision; frequent finding in surgical series
Type VI	Variable (Neurological)	Insertion into flexor retinaculum; potential tibial nerve compression	Consider MRI for tarsal tunnel involvement; selective excision if symptoms persist

**Table 3:** Surgical treatment stratified by plantaris tendon type.

Plantaris Tendon Type	Surgical Procedure	Clinical Notes
Type I	Open excision + possible Achilles debridement	Highest risk of conflict; frequently indicated in surgery
Type II	Resection (open or endoscopic)	Moderate risk; decision guided by imaging and symptoms
Type III	Endoscopic resection	Lower risk; may cause dynamic shearing under load
Type IV	No surgery—conservative management	No anatomical conflict; surgical intervention not indicated
Type V	Resection + debridement (often recommended)	Broad insertion; common in symptomatic refractory cases
Type VI	Resection + tibial nerve release	Rare variant; often misdiagnosed as tarsal tunnel syndrome

**Table 4:** Proposed treatment algorithm based on plantaris tendon type.

Plantaris Type	Conflict Potential	Recommended Treatment
Type I	High	Open excision ± Achilles debridement
Type V	High	Open excision ± Achilles debridement
Type II	Moderate	Endoscopic excision
Type III	Moderate	Endoscopic excision
Type IV	Low/None	Conservative treatment only
Type VI	Neurovascular conflict	Resection + tibial nerve decompression

## Abbreviations

Mid-AT	Midportion Achilles Tendinopathy
PT	plantaris tendon
AT	Achilles tendon
US	Ultrasound
MRI	Magnetic Resonance Imaging
ESWT	Extracorporeal Shock Wave Therapy
PRP	Platelet-Rich Plasma
HSR	Heavy Slow Resistance
TTS	Tarsal Tunnel Syndrome
